# Challenges and Opportunities in Developing Respiratory Syncytial Virus Therapeutics

**DOI:** 10.1093/infdis/jiu828

**Published:** 2015-02-23

**Authors:** Eric A. F. Simões, John P. DeVincenzo, Michael Boeckh, Louis Bont, James E. Crowe, Paul Griffiths, Frederick G. Hayden, Richard L. Hodinka, Rosalind L. Smyth, Keith Spencer, Steffen Thirstrup, Edward E. Walsh, Richard J. Whitley

**Affiliations:** 1 Department of Pediatrics, University of Colorado School of Medicine, and Colorado School of Public Health, Aurora; 2 Department of Pediatrics, Division of Infectious Diseases, and Department of Microbiology, Immunology and Biochemistry, University of Tennessee School of Medicine; 3 Children's Foundation Research Institute at Le Bonheur Children's Hospital, Memphis; 4 Department of Pediatrics and the Vanderbilt Vaccine Center, Vanderbilt University, Nashville, Tennessee; 5 Fred Hutchinson Cancer Research Center and University of Washington, Seattle; 6 Department of Medicine, University of Virginia School of Medicine, Charlottesville; 7 Clinical Virology Laboratory,Children's Hospital of Philadelphia, Pennsylvania; 8 Department of Medicine, Infectious Diseases Unit, Rochester General Hospital, New York; 9 Department of Pediatrics, Microbiology, Medicine and Neurosurgery, University of Alabama at Birmingham; 10 Department of Pediatrics and Laboratory of Translational Immunology, University Medical CenterUtrecht, The Netherlands; 11 Centre for Virology, University College London Medical School; 12 Department of Pediatrics, University College London Institute of Child Health; 13 Wellcome Trust, Innovations, London; 14 NDA Advisory Services Ltd, Leatherhead, United Kingdom; 15 Department of Health Sciences, University of Copenhagen, Denmark

**Keywords:** respiratory syncytial virus, therapeutic strategies, patient populations

## Abstract

Two meetings, one sponsored by the Wellcome Trust in 2012 and the other by the Global Virology Foundation in 2013, assembled academic, public health and pharmaceutical industry experts to assess the challenges and opportunities for developing antivirals for the treatment of respiratory syncytial virus (RSV) infections. The practicalities of clinical trials and establishing reliable outcome measures in different target groups were discussed in the context of the regulatory pathways that could accelerate the translation of promising compounds into licensed agents. RSV drug development is hampered by the perceptions of a relatively small and fragmented market that may discourage major pharmaceutical company investment. Conversely, the public health need is far too large for RSV to be designated an orphan or neglected disease. Recent advances in understanding RSV epidemiology, improved point-of-care diagnostics, and identification of candidate antiviral drugs argue that the major obstacles to drug development can and will be overcome. Further progress will depend on studies of disease pathogenesis and knowledge provided from controlled clinical trials of these new therapeutic agents. The use of combinations of inhibitors that have different mechanisms of action may be necessary to increase antiviral potency and reduce the risk of resistance emergence.

Respiratory syncytial virus (RSV) is the single most important cause of lower respiratory tract infection (LRTI) in infants and young children worldwide and can cause LRTI in elderly and immunocompromised patients; it is associated with significant morbidity and mortality in these target populations. No effective licensed therapies are generally available, but existing and emerging point-of-care diagnostics and investigational RSV-specific antiviral inhibitors offer promise of progress. In industrialized countries, RSV receives little publicity and is not widely recognized by the general public. There is a common, mistaken belief among drug developers that RSV is a disease primarily of preterm infants and that the disease is fundamentally different in term infants and older children. Furthermore, the adult market is assumed to be small. This underlines 2 of the fundamental obstacles that have constrained antiviral product development: perception of disease severity and clinical end points. This report summarizes outputs from 2 meetings addressing the challenges in developing RSV therapeutics.

## BURDEN OF RSV INFECTION

### Infants and Young Children

By the age of 1 year, 60%–70% of children have been infected by RSV (2%–3% of whom are hospitalized) [1], and almost all children are infected by 2 years [2]. RSV is estimated to cause 33.8 million cases of acute respiratory illness in children <5 years of age globally, resulting in 2.8–4.3 million hospital admissions and 66 000–199 000 deaths in 2005 [3]. Most infected infants experience upper respiratory tract symptoms, but 20%–30% will develop LRTI (bronchiolitis and/or pneumonia). Bronchiolitis, also known as RSV-associated acute LRTI, is frequently associated with coinfection with other viral agents, particularly human rhinovirus infections [4–8]. Signs and symptoms of disease include tachypnea, cough, crackles, and wheezing. Frequently, disease is associated with lethargy, irritability, and poor feeding. Comorbid factors, which increase the risk of severe LRTI, include prematurity, cyanotic or complicated congenital heart disease, chronic lung disease of prematurity, immunodeficiency, and immunosuppressive therapy (at any age for the last 2). Some children, including those otherwise healthy, are at risk for disease progression to respiratory failure, mechanical ventilation, and intensive care unit management. The incubation period ranges from 2 to 8 days, but 4–6 days is most common.

Because 99% of the deaths reported from RSV in 2005 [3] occurred in developing countries, reducing the burden of RSV infections has become a priority of the World Health Organization's new BRaVe (Battle Against Respiratory Viruses) initiative [9]. Ongoing population-based surveillance studies in several emerging countries will provide a platform to more accurately define the global burden of disease in children. Such data are beginning to emerge from Kenya and Malaysia, where life-threatening disease seems more common than in Europe and North America [10, 11].

Most infections occur between November and April in northern temperate locations [12]. In the United States, RSV is associated with 18% of all respiratory illnesses in children <5 years, 20% of all hospitalizations, 18% of all emergency department visits, and 15% of all pediatric office visits [1]. In subtropical regions RSV outbreaks tend to occur in cool seasons, either dry or wet, whereas in equatorial regions RSV tends to be detected throughout the year but with periodic increased activity [13]. The incidence and severity of disease in the developing world, including sub-Saharan Africa, are not well documented, but meta-analyses suggest that RSV is an important cause of death [3]. The timing of RSV outbreaks within a specific locale is usually quite similar from year to year; thus, local data are needed to more accurately predict timing of RSV outbreaks in a given community [14, 15]. After RSV bronchiolitis, some children develop a postbronchiolitic wheezing syndrome, but whether RSV-associated wheezing confers long-term risk for subsequent development of asthma remains controversial [16–23]. A meta-analysis of published data suggests a causal association between infant RSV hospitalization and respiratory morbidity (asthma/wheezing) that decreases with age [24].

### Effect of Illness on Parental Loss of Time From Work

Infection caused by RSV in adults was recognized approximately 2 decades ago [25–27]. There is an increasing recognition of the important burden of illness caused by RSV in adults, especially the elderly, in whom infection causes a broad spectrum of respiratory illness, ranging from colds to severe pneumonia. In ambulatory elderly persons, surveillance studies have found that about 5%–10% of those attending adult daycare facilities develop RSV infection annually [28]. Patients with stage 3 or 4 chronic obstructive pulmonary disease (COPD) and those with congestive heart failure are at particularly high risk, making them primary targets for treatment.

RSV is estimated to cause up to 10 000 deaths a year in the United States, most in the elderly population [29]. However, RSV infection cannot be differentiated clinically from influenza or from other respiratory viral illnesses in elderly or high-risk patients [4]. Studies using molecular diagnostics have found similar hospitalization rates and outcomes for influenza and RSV in older adults, especially those immunized against influenza [30–32]. Risk factors for hospitalization of RSV-infected adults include age >65 years, COPD, lower neutralizing antibody titer, radiographic evidence of pneumonia, bacterial superinfection, and lower functional status [5, 6, 32, 33]. Studies investigating the relative impact of influenza and RSV on hospitalization and mortality in elderly populations appear in Table 1, which shows that RSV can cause severe lower respiratory complications in older adults similar to influenza [7, 29–32, 34–39].

**Table 1.** Studies Investigating the Relative Impact of Influenza and RSV on Hospitalization and Mortality Rates in Elderly Populations

Overall design	Population	Location	Duration of Surveillance, y	Key Outcomes	Reference
Regression models to associate total death counts in individuals	Healthy elderly adults	The Netherlands	9	Mortality rates: influenza 1.6-fold higher than for RSV	[32]
Age-specific Poisson regression models using national viral surveillance data	Adults >65 y	United States	22	Mortality rates: influenza 3.7-fold higher than RSV	[24]
HMO databases used to estimate influenza- and RSV-associated hospitalizations	Adults who did not receive influenza vaccination	United States	3	Hospitalization rate for influenza twice rate for RSV; RSV rates were 4-fold greater in high-risk persons aged >65 y	[33]
Estimation of hospitalizations for RSV and influenza based on state hospital discharge databases	Approximately 40% of population	United States	15	Hospitalizations per 100 000 patient-years: 86 for RSV and 309 for influenza in patients aged >65 y; 12.8 for RSV and 65.6 for influenza in patients aged 50–64 y	[27]
Diagnosis-specific study using uniplex PCR	1471 hospitalizations	United States	4	Hospitalizations for RSV: 77 per 100 000 patient-years for patients aged >65 y	[34]
Rates of hospitalizations for RSV and HMPV compared with influenza	508 adults aged >50 y	United States	3	Hospitalization rates due to RSV, HMPV, and influenza: 15.01, 9.82, and 11.81 per 10 000 residents	[26]
Multicenter, case-control vaccine efficacy study	826 hospitalized patients	Spain	1	102 hospitalizations (12%) for influenza, 116 (14%) for other respiratory viruses	[35]
Retrospective cohort study	607 adults hospitalized with RSV	Asia	3	All-cause mortality: 9.1% at 30 d, 11.9% at 60 d	[28]
Smaller studies	…	Europe and Africa	1–3	Mean rate of hospitalization across smaller studies: 6.8% for RSV, 10.4% for influenza	[36–38]

Abbreviations: HMO, health maintenance organization; HMPV, metapneumovirus; PCR, polymerase chain reaction; RSV, respiratory syncytial virus.

Among working adults, respiratory illnesses caused by RSV can be protracted [40]. Compared with influenza, RSV infections more often cause nasal congestion, ear and sinus involvement, and productive cough but are less likely to cause fever, headache, and work absence (38% for RSV vs 66% for influenza) [40]. The occurrence of annual epidemics of RSV, the potential of RSV to reinfect all age groups, and the morbidity associated with these reinfections suggest that RSV infections in working adults result in appreciable costs for medical visits and work absenteeism. Furthermore, transmission of RSV in households is common, with approximately 40% of all family members >1 year old becoming infected [41], and provides an epidemiologic locus to test both chemoprophylaxis and treatment strategies.

### Immunocompromised Hosts

LRTI commonly follows RSV infection in many immunocompromised hosts, including children with immunodeficiency; hematopoietic stem cell transplant (HSCT) and solid organ transplant recipients (especially lung transplant recipients); patients receiving chemotherapy for leukemia, lymphoma, or cancer; and those with advanced human immunodeficiency virus (HIV) infection. RSV RNA detection in the blood may be a marker for lung injury and poor outcome [42].

HSCT recipients are at high risk of RSV infections that can result in LRTI, airflow obstruction, bronchiolitis obliterans syndrome (BOS), and death [43, 44]. Retrospective studies of HSCT recipients with RSV infection [45, 46] showed that 25%–29% develop LRTI. Risk factors independently associated with progression to RSV LRTI were older adult age, male sex, smoking history, lymphocytopenia, and conditioning with high-dose total body irradiation [47–49]. Similarly, independent risk factors for the development of LRTI and mortality for severely immunocompromised children were age <2 years, lymphocytopenia, and the presence of graft-vs-host disease [50]. In a 2013 study of 280 adult HSCT recipients with RSV infection, the 90-day mortality was 18% [45]. In another study of 118 adult HSCT recipients with RSV LRTI, the overall and respiratory failure-related 90-day mortality rates 47% and 34%, respectively [40]. Factors associated with increased mortality were bone marrow as stem cell source, oxygen requirement at diagnosis, the presence of copathogens, leukopenia, and high doses of corticosteroids (>2 mg/kg) [47, 49, 51]. Nosocomial transmission of RSV has been documented in an ambulatory care setting, with subsequent attendant morbidity [52]. Documentation of clonal strains within this setting highlights the importance of infection control intervention.

Immunocompromised patients with RSV may develop late-onset airflow obstruction, interstitial pneumonia, and wheezing. RSV and other community-acquired respiratory viruses are associated with BOS in lung transplant recipients [53–55]. BOS is a significant cause of morbidity and mortality in these patients, for whom retransplantation remains the only feasible treatment at this time. The incidence of BOS at 90 or 180 days after RSV infection has been estimated at approximately 30%–50%, with a 90-day mortality rate of 0%–12%. As noted above, this syndrome also occurs in HSCT recipients, but the exact incidence is not known [44].

## RSV PATHOGENESIS

### Infants and Young Children

As noted above, RSV disease in infants ranges from mild upper respiratory tract infection (URTI) to necrotizing bronchiolitis and pneumonia causing respiratory failure [56]. We know surprisingly little about the timing and events occurring in early RSV infections of infants; most of our knowledge has been developed after studying infants who are already hospitalized with infection [12]. Acute RSV bronchiolitis is characterized by extensive viral infection involving the ciliated respiratory epithelial cells, apoptotic sloughing of these cells into the alveolar and bronchial lumen, and recruitment of an accompanying cellular infiltration into the airway lumen, composed mainly of polymorphonuclear cells. Mononuclear cells can be identified as minority populations within these occluded airway lumens, but mucus and mucins contribute very little if at all to airway obstruction [57]. Polymorphonuclear cells are also present on histologic examination, as noted below.

Analysis of inflammatory responses from infants with fatal RSVLRTI identified inadequate (rather than excessive) innate and RSV-specific cell-mediated (CD8 and CD4) immune responses, robust viral replication, and apoptotic crisis [9]. In addition, naturally occurring genetic polymorphisms also implicate reduced innate immune responses as contributing to RSV disease severity [58–64]. RSV replication persists for a surprisingly long time in infants (≥2–3 weeks) and significantly longer than for similarly aged patients with influenza [65–68].

Although controversial at first, correlations between viral load and disease severity have now been established by multiple investigators using various techniques including quantitative culture and reverse-transcription polymerase chain reaction (RT-PCR). In RSV bronchiolitis, an excellent correlation exists between initial viral load in the upper and lower airways in intubated children (enabling access to lower respiratory tract secretions for testing) [69], thus making nasal viral load a useful, though not completely parallel, surrogate marker for lower respiratory tract viral load. Viral loads in the upper and lower respiratory tracts decline in parallel once peak viral load is achieved [69]. Studies of nasal viral load in naturally infected infants have shown that higher viral load collected shortly after hospital admission correlates with clinically meaningful outcomes, including longer duration of hospitalization, higher risk of requiring intensive care, and higher risk of requiring mechanical ventilation [67, 68]. In a multiple logistic regression modeled study of RSV dynamics, a more rapid rate of RSV clearance also correlated with more rapid clinical improvement in infants [58]. At this time, viral load remains the most important biomarker of RSV disease severity, but although it is an important factor in disease severity, other environmental or host factors are also significant [13, 58, 67, 68, 70].

Detailed analyses of cytokine and chemokine responses of infants with RSV bronchiolitis have identified a robust inflammatory response, which subsides as the infant recovers [71–73]. However, most analyses of these responses failed to also measure viral load, and those that did find strong correlations between higher viral loads and the inflammatory response markers measured [74]. It is unclear whether this immune response is appropriate and necessary for recovery or whether it contributes to more severe disease [75]. The multiple well-designed large studies showing insignificant benefit of corticosteroids in RSV infection suggest that those immune responses, which are steroid responsive, do not contribute significantly to RSV disease [76–78]. Although historical studies have addressed the role of the inflammatory response in pathogenesis, more recent studies suggest that deficiencies in immune responses [30, 31] might contribute to the severity of RSV disease in infants [79]. Host genetic polymorphism analyses seem unsuitable for the study of patient profiling, because disease susceptibility was estimated to be only 22% in one twin cohort study [80].

Small animal models suggest that acquired immune responses, in particular virus-specific T-cell–mediated responses, contribute to disease [81]. However, for children, low T-cell numbers clearly increase the risk of severe RSV infections [50]. Furthermore, T cells are not commonly found in the airway lumen or in the lung tissues of patients with RSV bronchiolitis [57, 82], and there is increasing skepticism about the ability of most small animal models of RSV to appropriately model RSV infection and disease pathogenesis in children. The neutrophil is the dominant inflammatory cell type identified in the lungs of children with RSV bronchiolitis, accounting for >80% of the inflammatory cells within the lumen of the lower airways [51]. The fact that neutrophils in the lungs of infants with bronchiolitis are highly activated suggests an important role in pathogenesis [79, 83], and when this role is better understood it may offer a potential target for host-directed therapies.

Risk factors for the development of severe disease include younger age [5, 84]. Most infants and children hospitalized with RSV were not born prematurely and ere previously healthy but were born close to the start of the RSV season and have high viral exposure through siblings or daycare [85]. As noted above, comorbid conditions, especially prematurity, congenital heart disease, and Down syndrome [6, 86] seem to increase the risk of disease severity. Environmental and demographic factors, such as crowding, daycare, and smoke exposure, are commonly listed in the literature as increasing the severity of RSV infections, but most of this increase, if not all, may simply be due to a heightened risk of exposure to the virus itself at an early age [59].

### Adults

In adults, the association between viral load and disease severity is less clear than in children, but this difference may reflect less attention to studying the disease in adults and the need for quantitative PCR techniques to quantify RSV load in this population [87]. A stronger association may exist between nasal levels of inflammatory mediators and disease severity [33]. However, prolonged detection of virus is demonstrable in adults hospitalized with RSV infection (mean, 13.1 days in nasal secretions and 10.1 days in sputa) [30, 33], suggesting that even delayed initiation of antiviral therapy might be beneficial. Bacterial coinfection, defined by classic microbiologic findings or implied by a serum procalcitonin level ≥0.25 ng/mL, has been found in approximately one-third of adults hospitalized with RSV infections. It is a significant problem, because it has also been associated with approximately 40% of viral respiratory tract infections requiring hospitalization [88], although studies in this patient group are complicated by multiple comorbid conditions and associated polypharmacy.

## ROLE OF DIAGNOSTICS AND MEASUREMENT OF VIRAL LOAD IN THE DEVELOPMENT OF RSV THERAPEUTICS

RSV is impossible to differentiate from other respiratory virus infections by clinical symptoms alone [89]; therefore, rapidly available results of laboratory diagnostic tests are required for enrollment into clinical trials. The increasing availability of nucleic acid amplification tests (NAATs), capable of detecting low concentrations of viral RNA, has transformed RSV diagnosis and is currently the laboratory diagnostic method of choice, especially in adults, in whom RSV loads are often significantly lower than the threshold of detection of most non-PCR assays. Because of higher RSV loads in infants and children, point-of-care rapid antigen tests, commercially available in various forms, are reasonably sensitive and specific and could be used for case selection in the context of a clinical trial or in routine RSV antiviral use. Table 2 summarizes some of the key characteristics of the tests available to detect RSV. (For more detailed information, please see references [90–96].)

**Table 2.** Tests Available for Detection of RSV^a^

Test	Sensitivity	Specificity	Clinical Usefulness
Virus culture systems^b^			
Conventional tube	Moderate	High	Reference standard for many years; slow, time-consuming, labor intensive
Shell vial/plate	Moderate	High	More rapid centrifugation-assisted culture; normally less sensitive than tube culture
Rapid antigen detection tests^c^			
Solid-phase immunoassay	Moderate	Moderate-high	Self-contained devices; rapid and easy to use; near-patient testing enabled; sensitivity can vary by test selected
Immunofluorescence assay	Moderate	Moderate-high	Rapid and normally more sensitive than solid-phase immunoassays; moderately complex and subjective reading; high sensitivity in hospitalized infants
NAATs^d^	High	High	New reference standard; superior performance characteristics; expense, labor, and time compatible; commercially available and highly automatable
Serology	NA	NA	Not useful for diagnosis; primarily used for epidemiologic studies

Abbreviations: NA, not applicable; NAATs, nucleic acid amplification tests; RSV, respiratory syncytial virus.

^a^ In general, the performance of selected tests for RSV may vary depending on the type and condition of specimen collected, the prevalence of the virus in a community, the time of year when testing is performed, and the patient population examined.

^b^ RSV is quite thermolabile and not easy to grow in culture. Cultures are most reliable in young children and less so in older children, adults, and immunocompromised hosts owing to shorter time of shedding, lower viral loads, and dry mucosa.

^c^ Antigen tests have moderate sensitivity compared with virus culture and even less sensitivity compared with molecular methods. Like virus culture, antigen tests are more reliable in younger children. Specificity may be significantly reduced when disease prevalence is low.

^d^ NAATs are more sensitive than any combination of culture- and/or antigen-based tests.

RSV quantification is central in monitoring response to experimental antiviral therapies and may help in examining the progression of infection, as well as the relationship between viral load and coinfections in acute respiratory disease [97–101]. Quantitative RT-PCR has been used to assess viral RNA load and is cost-effective compared with standard direct fluorescent antibody and culture [102, 103]. Viral load as measured by quantitative RT-PCR has been shown to correlate with viral load as measured by quantitative culture [67–69, 104] in children and adults. Furthermore, viral load as determined by quantitative RT-PCR has been shown to correlate temporally with the disease severity in experimentally RSV-infected adults [104]. The NAAT quantitative and qualitative analyses of specimens properly frozen and stored show little difference between fresh (nonfrozen) aliquots of the same specimen. Therefore, batching and central testing of clinical trial samples for diagnostic and assessment of antiviral effect is feasible. Likewise, genotypic analysis for resistance can also be accomplished with frozen specimens. It should be noted, however, that phenotypic analyses of the viruses collected in clinical trials is not generally feasible because of the limited ability of RSV to be cultured after a single freeze-thaw cycle.

## RSV PROPHYLAXIS AND TREATMENT: LESSONS LEARNED FROM PAST EXPERIENCE

Potential antiviral strategies for infection and disease include the following: prophylaxis, during the RSV season (seasonal) or after potential exposure (postexposure) to prevent illness and possibly infection; preemptive or early therapy, given when virus has been detected in the upper respiratory tract to prevent progression to lower respiratory tract involvement; and treatment of LRTI, initiated when virus is detected in specimens obtained from lower respiratory tract samples in patients with LRTI. The following sections briefly review key findings with these strategies in at-risk target populations.

### Infants and Children

Clearly, the most recognizable and clinically relevant treatment group is infants and young children with RSV bronchiolitis or LRTI. This population was the first for evaluation of therapeutic and prophylactic agents. The role of nonspecific interventions for the management of RSV LRTI was been summarized in a 2006 publication of the American Academy of Pediatrics [105] and will not be reviewed further here.

Seasonal prophylactic use of polyclonal RSV intravenous immunoglobulin (RespiGam) or human anti-F monoclonal antibodies (palivizumab, motavizumab) reduced the risk of RSV-associated acute LRTIs and hospitalizations in high-risk infants [106]. Palivizumab has become the accepted antibody product for prevention of RSV disease in at-risk children. It was licensed in 1988. Two Cochrane reports have summarized the use of palivizumab. The first assessed the data for disease prevention in infants. The data from 4 placebo-controlled trials demonstrated that active therapy decreased hospitalization (risk ratio, 0.49; 95% confidence interval, .37 to .64) compared with placebo [107]. Therapy was of greatest value in the premature and those at high risk (eg, congenital heart disease, chronic lung disease) Cost-effectiveness is greatest in this setting.

Currently, the American Academy of Pediatrics Red Book Committee recommends seasonal prophylaxis in the first year of life for infants of <29 weeks gestation. Prophylaxis is not recommended for healthy infants of >29 weeks gestation. In addition, those of <32 weeks gestation with chronic lung disease or heart disease or 32–35 weeks gestation with risk factors (daycare, siblings aged <1 year, smoke exposure in the home, congenital abnormalities of the airway) should be treated for 12 months. These recommendations will probably be further debated.

The second Cochrane Report analyzed the effect of palivizumab in children with cystic fibrosis ≤2 years of age. No meaningful differences in outcome were reported, albeit with small numbers [108].

A large prophylactic study comparing palivizumab with motavizumab in high-risk children showed that motavizumab was associated with a significant 50% reduction in the incidence of medically attended LRTI (MALRTI) compared with palivizumab, but also with a higher rate of adverse reactions [109].

Other studies compared the administration of palivizumab or motavizumab in infants hospitalized with established RSV LRTI and found inconsistent antiviral effect. Viral load was consistently reduced during these trials, as assessed by quantitative culture, [110], but this may have reflected ex vivo neutralization by the therapeutic antibody in the in vitro viral quantification, rather than reduced infection in the tissues of the patient [111]. A larger double-blind, randomized study of hospitalized infants treated with motavizumab or placebo demonstrated no antiviral effect, as measured by quantitative PCR [112], but the study was not powered to detect clinical benefit [111, 112].

Results of early studies of RSV prophylaxis with RSV intravenous immunoglobulin and palivizumab conducted in the United States [113], Canada, Europe [114, 115], and Japan [70] suggested that preventing RSV LRTI in high-risk infants also prevented subsequent recurrent wheezing up to 3 years of age. Similarly, a placebo-controlled trial in healthy preterm infants showed that palivizumab reduced the number of days of wheezing in the first year of life [116], even for wheezing not associated with an active RSV infection. If this association is indeed causal, as it seems, the development of an effective vaccine and perhaps a therapeutic(s) against RSV would have a major impact beyond the acute effects of RSV infection. Future studies of antiviral therapies should try to incorporate reduction in long-term wheezing as an outcome whenever feasible.

Ribavirin was first marketed in 1980 for treatment of RSV in children, but many of the original proof of concept studies, showing reduction in RSV load and its association with reduced disease severity, are considered flawed [117–124]. First, at that time, the only method to quantify RSV was by quantitative culture. Because aerosolized ribavirin is delivered in high concentrations to the same respiratory secretions from which samples were obtained for quantification, the observed reduction in viral load may have been at least partially attributed to an in vitro effect in the secretions due to the presence of drug. Second, in some studies, the placebo was aerosolized water, which is potentially bronchoconstrictive [124]. Third, efficacy outcomes were generally based on reductions in clinical severity scores which were of arguable clinical relevance. Furthermore, no ribavirin-induced mutations in RSV have been observed, which some (including the Division of Antiviral Products of the Food and Drug Administration [FDA]), interpret as suggesting a lack of a selective antiviral effect. Inhaled ribavirin has additional problems, including the potential for mutagenicity, teratogenicity, and carcinogenicity in preclinical models and the associated exposure risk to healthcare workers. Because of these occupational exposure issues and unclear efficacy, its use has been restricted to only very high-risk populations, namely HSCT and lung transplant recipients, as noted below, and replacement with selective small molecules is desirable [125].

### Immunocompromised Individuals of All Ages

Aerosolized ribavirin, with or without antibody treatment, has been commonly used in HSCT recipients, but no adequately powered randomized trials have been performed. One randomized controlled trial (RCT) compared preemptive aerosolized ribavirin to supportive care for RSV URTI in HSCT recipients but was discontinued after 5 years because of slow accrual, although trends toward reduced LRTI progression and nasal viral load reductions were observed in the ribavirin group [126]. Accrual problems in this trial were probably due to the complicated study design, which required masked clinical evaluations and the level of complexity caused by aerosolized ribavirin administration [126].

In retrospective cohort analyses, aerosolized ribavirin therapy reduced the risk of RSV LRTI by 83% and all-cause mortality by 57% in 280 HSCT recipients [45, 127]. Another recent study of 181 HSCT recipients with RSV URTI showed a trend toward less frequent progression to LRTI with aerosolized ribavirin [47]. In another uncontrolled and nonrandomized study, aerosolized ribavirin for the treatment of LRTI in 118 HSCT recipients was shown to be effective by multivariate analysis [42]. Systemic, namely oral, ribavirin showed trends toward moderate efficacy in uncontrolled observational studies, but the small sample size hampered statistical analysis. These results are consistent with results from a 2011 pooled analysis [43]. However, the concentrations of ribavirin achievable in humans by oral administration fall significantly below the concentration required to inhibit RSV replication (highlighting a major reason why ribavirin for RSV infection was developed as an aerosol) [128]. Ribavirin is deployed in high-risk immunocompromised children, as based upon adult uncontrolled treatment studies, as noted above.

Whether the addition of immunoglobulin products to antiviral therapy confers a significant benefit remains controversial. One uncontrolled retrospective cohort study (N = 280) suggested a benefit of the combination of immunoglobulin and ribavirin, but the relative contribution of the immunoglobulin products could not be evaluated [45]. In another study of HSCT recipients with RSV LRTI (N = 118) immunoglobulin products were not associated with a survival benefit [42]. Controversy also persists about the use of the RSV-specific monoclonal antibody palivizumab. A phase I study of aerosolized ribavirin plus palivizumab had an overall survival rate of 83% [129], but a follow-up phase III study was discontinued because of an inability to accrue patients owing to a perceived benefit of the interventions. Investigators conducting a retrospective uncontrolled single-center multivariate analysis (N = 84) concluded that the addition of palivizumab to aerosolized ribavirin did not improve outcomes in those with RSV-related LRTI compared with ribavirin alone [117]. In contrast, results of another study suggested that ribavirin, with or without an immune intervention, decreases progression to RSV-LRTI from 45% to 15% and death from 70% to 35% [127].

In RSV-infected lung transplant recipients, results of 2 double-blind, randomized, placebo-controlled trials suggest that early antiviral therapy can reduce the incidence of BOS compared with placebo. In the first RCT, involving 24 lung transplant recipients with RSV respiratory tract infection [130], inhaled Alnylam (ALN)-RSV01, a small interfering RNA targeting the N gene of RSV, lowered the incidence of new or progressive BOS at day 90 by 50% (*P* = .05). A follow-up RCT involving 77 RSV-infected lung transplant recipients demonstrated >50% reduction in new or progressive BOS development at both 90 and 180 days (intention-to-treat analysis, *P* < .05) [131, 132]. Preliminary results suggest that the treatment effect may be enhanced if ALN-RSV01 is given within 5 days of symptom onset, as opposed to later, although there were no significant differences in viral parameters or symptom scores during the acute phase of illness.

Only limited data are available on the association of viral load with outcome in immunocompromised patients. To our knowledge, there are no data on viral load in nasal secretions and progression to LRTI in transplant recipients. In a retrospective study of 30 HSCT recipients with RSV LRTI, no association was found with survival [42, 132, 133]. The lack of association of viral load and outcome in these studies may have been due to small sample size and the retrospective nature of the study, which prevented appropriate adjustment for bronchioalveloar lavage dilution effects. There was a higher probability of RSV RNA detection in serum samples from patients with higher viral load in bronchioalveloar lavage, but the effect did not reach statistical significance [42]. RSV RNA detection in blood has been associated with increased mortality in a study of HSCT recipients with RSV lower respiratory tract disease, and peak serum viral load above the median further increased the mortality risk [42]. Whether RSV RNA detection in blood represents active viral replication remains to be investigated.

## DEVELOPMENT PATHWAYS AND CHALLENGES FOR RSV ANTIVIRAL THERAPEUTICS

Several potent and selective RSV antiviral compounds have been identified in preclinical studies. Investment in development may be limited by a range of concerns, including an underappreciation of the burden of disease resulting in a misinterpretation of the potential market size, difficulties in RSV point-of-care diagnostics in select populations, particularly adults, and a previously held belief that RSV antiviral therapeutics would not work because the disease is driven primarily by virus-induced inflammatory cascades. Arguably, the medical need for antiviral development is greatest in young children, and safety is therefore of paramount importance. Major clinical challenges for other populations include relatively low infection rates in adult populations of interest and the uncertainty about the contribution of the inflammatory response versus viral load to RSV pathogenesis. Drawbacks with animal models and the historical ethical and safety concerns requiring demonstration of potential therapeutic benefit in adults before starting clinical trials in children, especially infants, have led to the use of experimentally induced RSV infection in otherwise healthy adult volunteers to study RSV pathogenesis and also to assess potential vaccines and therapeutic agents. In the human experimental infection model, viral replication kinetics seem to drive disease manifestations after RSV infection, an observation supporting a potential clinical benefit of RSV antivirals [104].

### Antiviral Targets

Fortunately, the technical barriers associated with antiviral drug development have been systematically overcome in the last decade, and there is little doubt that most of the tools required for product development are available. Most of the in vitro tools are well established, and, though in vivo models are still suboptimal, the development of the human challenge model has mitigated the early development risks. Most new agents have targeted the fusion protein, but other viral targets have been investigated and validated in preclinical studies. There is no lack of suitable antiviral targets.

The replication of RSV in vitro has been well studied. The RSV genome encodes 11 proteins, 3 of which contribute to the viral coat: small hydrophobic (SH), glycoprotein (G), and fusion (F) [134]. RSV is usually filamentous but shows great variation in form, the significance of which is unclear. Studies of the virion architecture show that particles range from 100 to 1000 nm in diameter and are spherical, filamentous, or a combination of both. Crystallographic structures have been produced for RSV M, N, and F proteins [135], and work is progressing on other RSV proteins, including M2–1, NS1, and NS2, and on replication complexes. In filamentous particles, the ribonucleocapsids are adjacent to an intermediate layer of protein assigned to M2-1 (an envelope-associated protein known to mediate association of ribonucleocapsids with the matrix protein) [136]. The RSV M protein is similar to that of filoviruses, suggesting that M-directed inhibitors might have activity across these viruses. Structure-based approaches will require structures with agents bound, while structures are not yet available for replication complexes.

Nucleolin has been identified as a receptor for RSV F protein [134]. Found mainly in the nucleus, it is also detectable at the apical surface of cells and acts as a molecular shuttle, moving material through intracellular compartments. This raises the possibility of drug repurposing (eg, of nucleolin-binding aptamers currently in phase II clinical cancer trials). The nucleolin inhibitor (G)-rich oligonucleotide showed dose-related antiviral effects in a mouse model of RSV infection [137].

A variety of steps in this replication cycle are potential targets for antivirals. Prevention of virus attachment to cells would be desirable; potential host attachment factors include glycosaminoglycans [138], the fractalkine receptor CX3CR1 [139], and nucleolin [140]. Induction of protein kinase R [141] and formation of host RNA stress granules [142] are required for efficient viral replication. RhoA plays an important but poorly defined role in RSV replication, and inhibition of RhoA function can decrease RSV replication [143, 144]. Assembly and budding of RSV in polarized epithelial cells occurs via a unique pathway that is independent of the multivesicular body apparatus and controlled by host proteins in the apical recycling endosome [145, 146].

Pharmacologic inhibition of fusion of viral membrane with cell membrane (mediated by the RSV F protein) is a common target. A newly discovered metastable prefusion confirmation state of RSV F glycoprotein was reported in 2013 [147]. It has a region that may be recognized by antibodies, making the virus vulnerable when F is present in its prefusion state. This specific site is named antigenic site “zero,” which is lacking in the postfusion RSV F glycoprotein. Indeed, highly potent neutralizing antibodies have been developed against antigenic site zero of the prefusion F glycoprotein [148]. The highly potent D25 RSV antibody has strong specificity for the prefusion F protein and stabilizes the protein in its vulnerable prefusion confirmation [149]. Stabilized prefusion RSV F is now being used to develop promising RSV vaccines as well as next-generation neutralizing antibodies for therapeutic usage.

RSV-induced pathology could be affected by targeting viral protein elements that trigger damaging inflammatory responses, such as the CX3C motif on G protein. Promising results have been obtained in mice using antibody-based agents to inhibit G protein signaling. Although monoclonal antibodies to G exert both antiviral and anti-inflammatory effects in mouse models [150], Fab fragments reduce inflammatory responses without inhibiting viral replication. The use of host-directed agents in concert with antivirals is also an appealing area for investigation, although there is a general reluctance to develop agents that interfere with host processes, owing to concerns about toxicity.

Characteristic morphologic changes occur in cells after RSV infection, including cytoskeletal rounding and cell sloughing into the respiratory tract. This process seems to be dependent on the NS2 protein and independent of this protein's recognized role as an interferon antagonist [151].

## ADVANCING SMALL MOLECULE THERAPIES

### Prophylactic and Antiviral Agents in Clinical Development

Molecules in development for the treatment of RSV are summarized in Table 3.

**Table 3.** Agents in Development for RSV Treatment^a^

Agent	Target	Notes
GS-5806	Fusion inhibitor	Oral RSV entry inhibitor
VP-14637	Fusion inhibitor	…
JNJ-2408068	Fusion inhibitor	…
MDT-637	Fusion inhibitor	Development on hold
ALX-0171	Fusion inhibitor	Nanobody technology
ALS-8176	RSV polymerase	Orally bioavailable
T-705 (favipiravir)	Influenza polymerase	Some activity against RSV
RSV-604 (A-60444)	N-terminal region of nucleocapsid protein	Effective both therapeutically and prophylactically in vitro

Abbreviation: RSV, respiratory syncytial virus.

^a^ Source: References [133–136].

#### Motavizumab

As noted above, the monoclonal antibody motavizumab has been evaluated in a study of 118 infants of whom 112 were confirmed to have infection. In this placebo-controlled treatment trial, no antiviral or clinical effect was determined, as noted above [112].

#### Fusion Inhibitors

The orally bioavailable F protein inhibitor GS-5806 has been evaluated in a phase I safety study and in the human challenge model. This molecule reduced both virologic and clinical outcome measures in the human challenge model. GS-5806 is advancing in clinical development, with plans for treatment studies in RSV-infected hospitalized adults and immunocompromised hosts [152].

Other small molecule inhibitors that interfere with RSV fusion through interaction with the F protein of RSV have also been identified, including VP-14637 (now reformulated for improved aerosol delivery and renamed MDT-637) and JNJ-2408068 [153]. The nanobody ALX-0171 is also in phase I development.

### Polymerase Inhibitors

Polymerase is another target of significant interest; polymerase inhibitors work prophylactically and also seem to have a large window of activity after infection, making them particularly attractive. ALS-8176 is a first-in-class nucleoside analogue targeting the RSV polymerase and is currently in clinical development. It too was very effective in the human challenge model [154]. Given the recent product launches for hepatitis C virus. this is an interesting approach and might confer the compound with a high barrier to resistance, as long as any safety issues can be negotiated. The anti-influenza agent T-705 (favipiravir) inhibits influenza polymerase and has some in vitro activity against RSV, but at higher concentrations than those needed to inhibit influenza [155].

RSV-604 (A-60444) is presumed to function by inhibiting viral replication via the N-terminal region of the nucleocapsid protein. This molecule was investigated both in stem cell transplant recipients with RSV infection and in the human challenge model. Clinical development is presumed to have been terminated, and unfortunately no data on the clinical outcome have been published to date to our knowledge.

### Preclinical Hurdles

#### In Vitro Assays

Cell-based screening is likely to be more productive than structure-based approaches in the short term, but such screens have mostly identified fusion inhibitors. Replicon systems that target viral replication and the polymerase complex also offer the potential for development of high-throughput screening assays [156]. RSV infection can be studied in human upper respiratory tissue explants, but the expense, source variability, and other logistical considerations preclude their routine use in antiviral discovery. There may be advantages to using cultured human cells.

#### Animal Models

Since the initial description of RSV as a chimpanzee coryza agent, both small and large animal models of RSV infection have been used [157], but all have significant limitations with regard to assessing the effects of antivirals. One general problem is the lack of standardization in inoculum preparation and assays of outcome measures across different laboratories. It is difficult to obtain purified preparations of virus, because other constituents present in the preparations could affect responses.

Mice (including transgenic and knockout strains) are not natural hosts, high intranasal viral inocula are required, viral replication is minimal, and spread of infection through the lungs is not the same as in humans; both pathogenic and functional immune mechanisms are also not necessarily the same in animals compared with humans. Animal models using juvenile mice have been developed and offer some advantages in that they seem to parallel human disease somewhat better than more established models of older rodents [158–163]. Additional work is needed in juvenile and aged mice, for both primary infection and reinfection. Because of its relative lack of ability to mount an interferon response, the cotton rat has also been widely used to study RSV infection and disease [164]. Viral kinetics in the cotton rat model parallel those in humans more than mouse models, but disease still does not sufficiently parallel events in the infant disease. Guinea pigs and chinchilla have also been used on occasion, the latter being popular for otitis media studies.

Nonhuman primate models provide the closest model for testing vaccines and antivirals, because chimpanzee infection more closely resembles human disease. Bonnet monkeys, rhesus macaques, African green monkeys, and baboons have also been used. However, most of these primates are no longer readily available (chimpanzee and bonnet monkey), and large breeding colonies, required for testing therapies in infant primates, are limited. Bovine RSV has been studied in cows, and a sheep model using bovine RSV is popular for studying neonatal biology. The pathology seen in neonatal sheep models seems similar to human infections. However, given the limitation of all of these models as applied to humans, a human challenge model has been developed, as discussed below.

#### Experimental Human Infections

Human challenge studies are used to obtain proof-of-concept efficacy and plan clinical trials in natural RSV infection. Indeed, such studies have been performed since 1971 [165]. Limited experience indicates that they seem predictive of field observations with RSV interventions, having been used in the clinical development of aerosolized ribavirin. Humans can be infected with low quantities of standardized inocula and offer controls, minimize confounding variables, and allow the study of both the host immune response(s) and viral kinetics, under carefully controlled conditions. Disadvantages include the fact that subjects are young, healthy adults with variable preexisting immunity. Furthermore, the studies are expensive (eg, quarantine is required).

At present, human challenge depends on access to the ultralow-passage clinical strain Memphis 37, manufactured according to good manufacturing practices. This strain is now controlled by a company, Retroscreen, and is currently approved for use only in the United Kingdom, which creates an obvious obstacle to conducting studies. There is an urgent need for the development of good manufacturing practice–qualified challenge viruses. The development of RSV challenge strains has recently been attempted in the United States, but as with vaccine manufacturing requirements, the FDA has oversight of the challenge viruses. These manufacturing specifications require passaging the virus many times in the FDA-approved Vero cell line, which probably reduces human infectivity. Additional challenge strains for human models are being developed at the National Institute of Allergy and Infectious Diseases, National Institutes of Health. Notably, the FDA finds it acceptable to review data generated from the Memphis 37 challenge model conducted outside the United States.

Experimental infection of volunteers has been used in the past to assess several candidate antivirals, including interferons [166]. More recently, the model was used to assess RNAi technology for future use against RSV and perhaps other viruses [167]. Among 88 healthy adults randomized 1:1 to ALN-RSV01 or placebo given both before and after viral inoculation, the proportions of culture-defined RSV infections were 71.4% and 44.2% in placebo and ALN-RSV01 recipients, respectively (*P* = .009), representing a 38% decrease [167]. This positive signal led to the subsequent testing of ALN-RSV01 in RSV-infected lung transplant recipients, but the molecule is not being advanced in clinical development [168].

A study of GS-5806, a novel small molecule fusion inhibitor, has been conducted in healthy adults [152]. It demonstrated that even a single dose of the drug was sufficient to abrogate viral replication in the healthy adult volunteers. Perhaps more importantly, it demonstrated that reduction in viral load after an RSV infection had already started and rapidly reduced disease severity. Similarly, as noted above, ALS-8176 was also active in this model and has been advanced into pediatric studies [154].

In evaluating the potential usefulness of antivirals for RSV, it is helpful to compare the viral and disease dynamics of experimental RSV and influenza infections and their respective treatment timing windows. Comparing viral and disease dynamics in healthy volunteers infected with either influenza or RSV suggests that reducing RSV load, if timed to coincide with clinically effective influenza antivirals, would provide a similar or greater window of opportunity to reduce clinical RSV disease [169].

#### Clinical Development Pathways

Virologic end points are required to establish that the drug has an antiviral effect, and to assess development of resistance. After demonstration of antiviral activity in experimentally infected volunteers, clinical end points are needed to detect meaningful patient-oriented benefits. Scoring systems have the highest power to detect clinical differences between groups. Trial designs should include a placebo control to account for variability of disease over time.

Each of the study populations presents their unique problems as well as potential benefit. Thus, the infant population has the greatest promise, because RSV is well recognized by pediatricians, it is easy to diagnose with rapid testing, and the market is sizeable. For adults, RSV is generally much less recognized by physicians and rapid diagnosis is not routine, so enrollment of adults for treatment studies is more problematic. Nevertheless, with improving diagnostics and the recognition of mortality in the elderly, implying clinical need, therapies will be evaluated in these populations. Options for future clinical studies, and the pros and cons of each, are outlined in Table 4.

**Table 4.** Options for Phase IIb and Phase III Clinical Studies in Different Populations

Population	Pros	Cons	Potential End Points
Children			
Outpatient	Easy to do; high prevalence (1:8–10 infants annually have RSV illness); the younger the patient, the higher likelihood of a severe outcome experience with Tamiflu/ACV in the outpatient setting; an antiviral is most likely to show an impact in this population	Large study needed, especially if more severe end points are chosen; difficult to standardize definitions; rapid diagnostics needed to identify children in the clinic; the shorter the duration of signs and symptoms, the more likely to show an impact and the more difficult to enroll patients	Prevention of LRTI, ED/urgent care visits/hospitalization, MALRTI, and acute otitis media
Emergency room visits	Large population; ER groups provide patient access	Disparate admission criteria (eg, to short-stay units, home oxygen use, hospital); population using ER might not represent severity of illness rather access to care; costly; multiple studies ongoing, with competition for patients	Hospitalization; duration of stay
Hospitalization	Captive population; most are hypoxemic; studies relatively easy to do	May be too late for an antiviral to show an impact; hospital stays are short, so it is difficult to demonstrate significant reductions; cost reduction may be difficult to demonstrate; some hospitalizations for social reasons	Duration of hospitalization and oxygen use; economic benefit; duration of ICU stay
ICU	Smaller study; fewer sites needed; more controlled environment; outcomes can be quantified	Probably too late to show antiviral effect; 1 outlier can affect mean values; when to stop treatment is undefined; ICU and NICU can have different criteria for discharge	Duration of ICU stay, ventilation, total hospitalization, and oxygen use
Premature infants	Small populations; relatively well defined; 2-fold higher rates of severe disease; infants of 32–35 wk gestation a possible group for placebo-controlled trials	Small population for treatment trials; many receive palivizumab (highest risk), so trials of prophylaxis will need an active control; infants of 32–35 wk gestation not seen as high-risk group in some areas of the world	Prevention of LRTI, ED/urgent care visits/hospitalization, and MALRTI
Congenital heart disease	Small populations; Relatively well defined; select groups have a higher rate of severe disease	Many receive palivizumab; surgical correction is occurring earlier and earlier, so risk is decreasing; heterogenous group: with or without pulmonary hypertension, right-to-left or left-to-right shunts, cyanotic or acyanotic	Prevention of LRTI, ED/urgent care visits/hospitalization, and MALRTI
Adults			
COPD	Population might vary from country to country; easy to do studies in these patients; placebo-controlled trials possible; CHF might be a risk factor for MALRTI in subjects with COPD	Possible RSV persistence; burden not completely established; only 2 longitudinal studies so far; low attack rates and burden; causative role in exacerbations might be difficult to establish	Prevention of MALRTI and exacerbations
Elderly	Huge population; relatively easy to do studies in this group; placebo-controlled trials possible; mortality rate in this population could make it very important to study	Disease not generally recognized by internists; no clear clinical syndrome (eg, bronchiolitis); overlap with influenza season; lack of a good rapid diagnostic (PCR needed for diagnosis); population-based studies in large centers might not be able to capture severe end point	Prevention of MALRTI and death
Immunocompromised			
Children	Small populations; relatively well defined; high rates of severe disease; easier to perform studies; no need for advocacy because mortality risk from RSV is well recognized	Many receive palivizumab (off label); many are given toxic drugs; possible drug-drug interactions; underlying conditions may dictate course physicians tend to intervene early with off-label treatments; immunosuppression varies and might determine outcome; RCTs more difficult to perform in these populations	Prevention of progression from URTI to LRTI, ED/urgent care visits/hospitalization, and death
Adults	Relatively large populations; high rates of severe disease; easier to perform studies; No need for advocacy Mortality Placebo-controlled trials possible?	Many receive palivizumab (off label); for prophylactic trials involve weight-based dosing, which is extremely costly; first 100 d most important (might not fall into RSV season); many receive other toxic drugs; drug-drug interactions	Prevention of progression from URTI to LRTI; prevention of ED/urgent care visits/hospitalization; prevention of death

Abbreviations: ACV, acyclovir; CHF, congestive heart failure; COPD, chronic obstructive pulmonary disease; ED, emergency department; ER, emergency room; ICU, intensive care unit; LRTI, lower respiratory tract infection; MALRTI, medically attended lower respiratory tract infection; NICU, neonatal ICU; PCR, polymerase chain reaction; RCTs, randomized controlled trials; RSV, respiratory syncytial virus; URTI, upper respiratory tract infection.

##### Clinical Development Pathways

RSV hospitalization occurs in infants and children a mean of 4 days after illness onset. Intuitively, antiviral therapies are likely to show clinical benefit if they are instituted early on in the course of the infection. This necessitates the evaluation of patients before hospitalization. Unfortunately, early outpatient RSV disease has not been studied. It is not yet known when peak viral load occurs with respect to the onset of URTI symptoms in infants attending outpatient clinics, or whether early detection of RSV infected outpatients is even possible before attaining peak viral loads, so that an intervention can be effective in reducing hospitalization. Such a biomarker requires evaluation. Detailed studies to define these viral and disease dynamics in infants and children are currently underway (John DeVincenzo, personal communication). In hospitalized infants <2 years of age, the rate of RSV clearance was an independent predictor of disease resolution and hospital discharge [58]. Moreover, URTI viral titers in nasal aspirates were significantly associated with subsequent development of respiratory failure and the requirement for intensive care. The therapeutic implication is that use of antiviral agents early in the disease course, even when viral replication is at or past its peak, might improve subsequent morbidity.

The number of medical office visits and emergency room visits for RSV infection are both many-fold higher than the rate of hospitalizations in pediatric populations, suggesting a much larger study population for interventions (Table 5). Because children <6 months of age have the highest rate of hospitalization, treatment studies should be designed to prevent hospitalization. However, the disadvantage of this end point is the large sample size.

**Table 5.** Visits to Different Healthcare Facilities for RSV Infection by Age Group^a^

Type of Visit	Visits per 1000 Children by Age Group			
	0–5 mo	6–11 mo	12–23 mo	24–59 mo
Hospitalization	11.7–21.7	3.4–7.4	1.9–3.2	0.2–0.4
ER visit	39–69	45–68	24–38	11–15
Practice visit	108–157	160–194	53–80	31–77

Abbreviations: ER, emergency room; RSV, respiratory syncytial virus.

^a^ Source: Hall et al [1].

Additional considerations that have not previously been explored include determining the impact of therapy on prevention or resolution of otitis media, as has been well documented in pediatric oseltamivir studies [170, 171], and loss of parental work time.

Impacts on long-term outcomes after RSV infection have the potential to change the cost-effectiveness of a treatment, so follow-up of treated patients is important. Specifically, long-term outcomes in treatment studies have been reported in studies linking RSV LRTI in infants with subsequent increased healthcare utilization and cost [172], reduced quality of life [173], recurrent wheezing during childhood [20, 172, 173], and asthma [172, 173]. As noted above, preventing RSV with RSV intravenous immunoglobulin [113] or palivizumab [70, 114, 115] significantly improved both respiratory outcomes as well as lung function studies up to 10 years later [113]. However, long-term follow-up studies of ribavirin did not show similar benefit [174].

##### Adult Populations

The burden of RSV disease in adult patients with COPD, asthma, or cystic fibrosis is incompletely understood, and RSV is only one of multiple community-acquired respiratory viruses, causing exacerbations of these conditions. As noted above, the problems of targeted antiviral studies in elderly persons have been iterated. Furthermore, the elderly are a heterogeneous population encompassing wide ranges in functional status and the presence and severity of comorbid conditions. The RSV season overlaps with the influenza season, which emphasizes the importance of NAAT-based diagnostic testing. Overall, the settings, entry criteria, and outcomes are very different between adult populations and children.

The burden of RSV mortality in the elderly represents a clear unmet medical need. However, community-based studies will be difficult in this population, because annual attack rates are low (3%–7%), requiring the screening of large numbers of ill individuals. Nursing homes provide an attractive alternative setting, providing an opportunity for surveillance and therapeutic intervention or prophylaxis. However, in such settings one would have to implement and provide relatively rapid nucleic acid amplification on site, because RSV diagnosis in adults is unreliable with the widely available and relatively inexpensive rapid antigen detection methods. Effective treatment in hospitalized patients is a medical need, but the reasons for hospitalization are heterogeneous and often related to comorbid conditions. Although it may be thought too late for antivirals to be effective, prolonged detection of RSV in respiratory secretions in hospitalized adults [33] suggests that intervention might benefit some patients, as has been established for antiviral therapy in hospitalized patients with influenza [175].

##### Immunocompromised Hosts

The high morbidity and substantial mortality of RSV illness in HSCT and lung transplant recipients makes them obvious candidates for evaluating new antiviral therapeutics. However, because of the very small number of these infected patients, mortality from non-RSV causes, the presence of multiple comorbid conditions, and the use of nonapproved RSV interventions, therapeutic studies in these populations pose challenges. Furthermore, because the incidence of infection ranges between 2% and 17% in such patients, studies would require multiple centers and extended periods of surveillance and enrollment, which could hamper rapid accrual of sufficient patients necessary for achieving proper statistical power. It is likely that placebo controls will be unacceptable; thus, alternative study designs (ie, adaptive trials) will need to be considered.

#### Antiviral Study Design Considerations

##### Prophylaxis

An obvious strategy for antiviral prophylaxis would be prolonged seasonal use in immunocompromised hosts or other high-risk groups. However, given the low attack rate combined with the limited patient population, this approach would pose significant challenges. A randomized, double-blind prospective design could be used in transplant recipients, but in at-risk infants ethical issues would necessitate comparing a study drug with palivizumab if the study population was the same as for the licensed product. However, if the population is different (eg, term infants born during the RSV season and <6 months of age, a population for which no vaccine or prophylaxis is available), placebo-controlled trials would be possible. Primary end points would include reductions in medically attended LRTIs and RSV-related hospitalizations.

A second strategy could take advantage of intrafamilial RSV transmission patterns to examine postexposure prophylaxis [41]. Household studies could identify outpatients or hospitalized infants with RSV and then follow family contacts, allowing confirmation by NAAT of intrafamilial transmission. Although an unproven trial design for RSV infection, it has been used successfully in influenza studies. Such a study is potentially much less expensive than standard prophylactic designs, and the same design could be used for early therapy trials.

##### Clinical End Points

The issue of clinically relevant end points in both children and adults has complicated the ability of industry to innovate. The selection of end points is of paramount importance; those in industry may feel cautious about developing a product against a particular clinical end point in one population that is not relevant to another population, thus requiring significant additional investment. Although some investigators believe that a reduction in viral load is sufficient, the lack of a clear correlation between disease severity and viral load in infants and adults means that a robust clinical end point must be agreed on. There is a need for additional natural history studies to help define end points for therapeutic studies.

In children, prior prophylaxis studies used RSV hospitalization as the primary end point. RSV hospitalization rates within these trials approached 10% when evaluated over the entire RSV season. It is problematic to extrapolate this hospitalization rate to what might be seen in treatment studies. The percentage of infants and children presenting to an outpatient facility with RSV and then requiring hospitalization has not been measured well. However, a small study conducted >20 years ago found that 8 of 55 infants <6 months of age presenting to an outpatient clinic with RSV were hospitalized. Of such infants <15 months of age, 11 of 127 (8.7%) were hospitalized [176]. Therefore, powering a phase 3 trial using hospitalization as an end point might be feasible but would require a potentially large study. A second clinical end point that has been proposed in the motavizumab versus palivizumab studies was MALRI, which has been used in the context of a large study of motavizumab with >330 sites in many countries in North America, Europe, and Australia [109]. MALRI includes physician visits, urgent care, emergency visits, and hospitalization and has been used or considered by many companies and the FDA as a clinical end point. However, MALRI as an end point may be seen as too subjective and dependent on cultural differences between patients in different countries. For example, in some countries, parents may seek attention for mild disease in their children, whereas in others the norm would not be to seek medical attention early. Clearly some component of MALRI will be used as an end point.

In adults the end points are less apparent, because respiratory hospitalizations might not be sufficiently frequent, except in elderly or immunocompromised hosts. MALRI, or some component of it, might be less useful, because adults often have cardiorespiratory comorbid conditions that could be the main reason for hospitalization, adding another layer of complexity in assigning causality to the end point.

##### Laboratory End Points

With several new small molecules entering clinical trials, it is possible to collect biologic specimens to assess for viral load and host response. Compartmentalized viral load may be a biomarker that identifies children at risk for progressive disease and, perhaps, with greater need for experimental combination therapy. Host response parameters may allow for advances in vaccine development.

##### Resistance

Irrespective of the investigational antiviral, preclinical and clinical studies must incorporate monitoring of resistance emergence with appropriate phenotypic and genotypic assays. Although resistance emergence is expected, especially when protracted replication occurs in the setting of insufficiently potent drug concentrations or severely immunocompromised patients, characterization of variants with amino acid substitutions in target proteins are needed to assess their effects on viral fitness (replication, virulence, transmissibility).

##### Industry Considerations

Although RSV is associated with a significant disease burden, the pharmaceutical industry usually requires a solid commercial foundation to invest in the development of new treatments. In the current climate, industry has focused on the large antiviral markets, such as hepatitis C virus, hepatitis B virus, and HIV, which require prolonged courses of treatment. In comparison, until recently, RSV therapeutic efforts have seemed fragmented and attracted sporadic interest from major companies.

Market estimates are difficult to precisely define and clouded by uncertainty in terms of both patient population and likely reimbursement. However, some analysts believe that the market for premature infants or infants with preexisting cardiopulmonary disease is not the key, because the majority of hospitalized cases of severe RSV infection are children who are not in high-risk groups, indicating that there is an underserved market in need of new interventions. There is also reasonable evidence that RSV plays a role in childhood asthma [116] and that preventing RSV can reduce the incidence of childhood wheezing. Therefore, the underserved market in children alone is significant. The cost of palivizumab has prevented its adoption in many groups of children who would benefit from prevention of RSV by the antibody, particularly in some parts of the world, but this should be viewed as an opportunity rather than a limitation. Because palivizumab is significantly more expensive to use in adult populations (because of adults' higher body weight), a product that could be used in both patient groups would satisfy market expectations. Currently, high-risk adult groups are completely neglected and denied the potential benefits of RSV passive antibody prophylaxis, representing a medical opportunity. If other patient groups with other respiratory complications (eg, COPD) can be accessed, then the market expands even further. The key issue for industry is identifying a product that can provide benefit to the majority of patients of all ages. A direct antiviral approach has the potential to cover all patient populations or patient groups not addressed by future vaccination.

Estimates for the adult market vary widely, from $500 million to >$2 billion. Such uncertainty is not unusual but at the same time generates a level of doubt in comparison with other potential markets that industry may enter. Along with the development challenges and regulatory uncertainty, this has led to many players remaining out of the field until a pathfinder molecule demonstrates the appropriate path to market. Ironically, any pathfinder molecule that reaches the market may take most of the profit, so the reality for industry is that there is a significant first-mover advantage.

### Regulatory Hurdles and Opportunities for Future Pediatric Therapeutics

RSV disease differs between children and adults is different. It is noteworthy that only a handful of categories of drugs have ever been developed primarily for pediatric patients (as opposed to being developed first in adults and then assessed for safety in the pediatric population in diseases that are similar in adult and pediatric populations). These include the following:

- Vaccines, with a different but well-trodden regulatory pathway
- Drugs for inborn errors of metabolism, with development stimulated by orphan drug designations given in the European Union and United States, by other regulatory agencies, and in close partnerships with National Institutes of Health laboratories
- RSV prophylactic antibody preparations (eg, RespiGam [RSV intravenous immunoglobulin], palivizumab)
- Surfactants (If regulatory approval had been attempted first in adults, it is unlikely that surfactants would have been approved for use in neonates, where they have saved thousands of lives.)

Clinicians, regulatory authorities, and public health experts are all interested in facilitating development of RSV antivirals for pediatric use. In the current ethical and US regulatory environment, evidence of safety and likely benefit to the individual potential pediatric study subject must be available before RSV antivirals are investigated in infants and very young children. It is widely assumed that at least safety studies need to be carried out in adults before children. Because adult disease is quite different from that in infants (with differences in viral load, clinical manifestations, comorbid conditions, and end points), phase III trials in children should not be dependent on a prior efficacy study in naturally infected adults. The type of adult data required before initiating pediatric development is not well defined. For example, antiviral efficacy data from the human RSV challenge model might be appropriate to provide proof of principle in concert with sufficient adult safety data to initiate studies in infants. Indeed, this seems to be the case, in that the FDA has allowed at least the ALS-8176 molecule to proceed directly into clinical evaluation in children. Because these would be early in children studies, they would likely be in older children first, then progress to younger children who are at greater risk.

Regulatory authorities in the United States will consider, among other factors, whether the following data would be sufficient to justify the initiation of RSV studies of a new agent in infants, assuming that the agent did not raise preclinical or clinical safety concerns and that there would be a prospect of direct clinical benefit from the intervention:

- Substantial evidence of safety according to good laboratory practice toxicology studies (including appropriate toxicology studies of juveniles)
- No clinically important safety findings observed in phase I studies in adult healthy volunteers evaluating exposures similar or higher than the weight-adjusted exposures to be evaluated in infants
- Demonstrated relevant pharmacokinetics/pharmacodynamics in a model—animal or human

Because most children ≥2 years old do not have severe enough RSV disease to warrant hospitalization, regulatory bodies regard as inappropriate the use of healthy older children as RSV therapy study subjects. However, younger children and infants generally have high enough risks for severe RSV disease to be able to justify enrolled in such studies.

Both an initial pharmacokinetic assessment and modeling would ideally be required for dose selection and movement directly into a treatment study in infants, but to some extent this might depend on the properties of the drug. For example, single-dose data might be sufficient if the drug has linear pharmacokinetics. Each new agent is likely to be reviewed by regulatory bodies on a case-by-case basis, with final judgments predicated on proper assessment of risk-benefit ratio.

Developing a safety database for a new therapy in adults before its introduction to children is helpful, although appropriate juvenile toxicity data will also be needed. Experimental infection model data may augment this safety database and suggest relevant treatment timing windows, to identify timing that will mimic the transition from URTI to LRTI in children. Whether a safety database in hospitalized infants is needed before initiating outpatient studies in infants has not been defined by regulatory bodies. In all likelihood it will be determined by animal toxicology and adult safety studies of individual experimental agents and will probably depend on the mode of action of the new agent, its route of administration, prior safety signals, the robustness of the safety analyses, the potential for the development of resistance and oversight of the pediatric protocol itself, and the population under study.

The number of subjects needed for a safety database for either prevention or treatment of RSV in infants to gain approval would depend on whether the indication was for prophylaxis or treatment. The regulatory pathway for RSV passive antibody prophylaxis (RSV immunoglobulin and palivizumab) first involved phase I studies in adult healthy volunteers and then immediately entered phase I studies in high-risk infants dosed during the respiratory season, followed by pediatric phase 2 and 3 studies. Phase IV studies in targeted patient populations will be a critical issue, especially when administered to otherwise healthy infants and young children. When treating patients with a lower risk of disease, the safety bar is higher. Conversely, a product with a higher level of toxicity may be acceptable if it shows benefit in severe disease.

There is no compelling need to study a new agent in the elderly or immunocompromised first, regardless of whether these groups are eventual target indications. Approval to evaluate new agents in the adult population would probably be easier to obtain, because there are fewer safety concerns. Studies could be done in parallel with those in children.

Finally, the cost-effectiveness of any treatment is important for the final implementation of future RSV therapeutics. Cost-effectiveness models are notoriously difficult, and RSV is no exception, not only for antivirals but particularly for prevention. The pharmaceutical industry appreciates that their future market depends to a large extent on the cost-effectiveness of the drug.

## CONCLUSIONS

RSV infections are a leading cause of acute and chronic disease in children and adults. Compared with those for HIV and hepatitis C virus, industry explorations of therapies for RSV infections have been limited. Several potent and selective antiviral compounds have been identified but generally have yet to pass through phase II trials to show antiviral evidence in humans. Development may have been slowed by a range of mistaken assumptions and dogma, for example, about the size and fragmentation of the potential market, the presumed difficulty of diagnosis, and the unfortunate previous belief that antivirals will not work because RSV disease is driven by delayed host inflammatory processes. The complexity of the route to market and the lack of a pathfinder are also potential obstacles. On the other hand, the absence of an existing RSV antiviral therapy and the large unmet medical need provides opportunity and the freedom to establish new pathways of evaluation and study.
